# Facial Involuntary Movements and Respiratory Failure in CANOMAD, Responsive to IVIG Therapy

**DOI:** 10.1155/2015/170543

**Published:** 2015-11-30

**Authors:** Kate Johnson, Ashish Malkan, Mohamed Shaffi

**Affiliations:** Department of Neurology, Nepean Hospital, Derby Street, Kingswood, NSW, Australia

## Abstract

CANOMAD is a rare chronic neuropathy, characterized by chronic sensory ataxia and intermittent brain stem symptoms due to antidisialosyl antibodies. The disorder results in significant morbidity but is poorly understood and often misdiagnosed. We describe a unique case of CANOMAD, associated with involuntary movements of the face; patient reported exacerbations with citrus and chocolate and respiratory muscle weakness. Our patient was initially misdiagnosed with Miller Fisher Syndrome, highlighting the need for vigilance should neurological symptoms recur in patients initially diagnosed with a Guillain Barre variant. Moreover, the optimal treatment is unknown. This patient responded remarkably to intravenous immunoglobulin and has been maintained on this treatment, without further exacerbations.

## 1. Introduction

CANOMAD (chronic ataxic neuropathy, ophthalmoplegia, IgM paraprotein, cold agglutinins, and antidisialosyl antibodies) is a rare chronic neuropathy, with approximately thirty cases described in the literature [1]. The disorder is characterized by chronic sensory ataxia and arreflexia with relatively preserved power in the limbs [1]. Additionally, patients commonly suffer relapsing, remitting brain stem symptoms, including ophthalmoplegia, dysphagia, and dysarthria [1, 2]. The course typically extends over decades but is highly variable [1]. Serologically, CANOMAD is defined by the presence of IgM antidisialosyl antibodies, typically to GD1b, GD3, GT1b, and GQ1b [2]. These antibodies are often cold agglutinins [2]. The symptoms of CANOMAD are highly disabling, but diagnosis is often delayed due to a number of factors. Physicians often fail to recognize the syndrome as patients may not develop the complete set of features for decades. There is also significant overlap with the Miller Fisher Syndrome (MFS) [3, 4]. The ideal treatment for CANOMAD remains unknown [1, 5, 6]. We describe a patient with CANOMAD who responded well to intravenous immunoglobulins (IVIG) and has been maintained on this treatment, without further exacerbations for over 12 months, providing support for the use of this immunomodulatory therapy for CANOMAD.

## 2. Case Presentation

A 68-year-old man was hospitalized following a fall. His background included sixteen years of ataxia and paresthesia of his limbs. Over the years numbness in his extremities gradually increased and walking became difficult, especially in darkness, resulting in falls. He had intermittent, spontaneously resolving diplopia. Five months earlier, a neurological deterioration necessitated mechanical ventilation. He was treated for MFS, responding to plasmapheresis. Interestingly, the patient reported avoiding citrus and dark chocolate as they exacerbated his neurological symptoms, as did major stressors, such as surgery. He had been treated for monoclonal gammopathy of unknown significance for several years.

Five days into this admission, the patient reported lip tingling, dysphagia, and dysarthria. Subsequently his ataxia worsened and he developed limb weakness. Examination revealed choreoathetoid movements in all four limbs as well as involuntary choreiform movements of facial musculature and tongue. The facial movements appeared nonpurposeful, irregular, and arrhythmic, seeming to flow from one part of the face to the other. The facial and buccolingual movements were present at rest and were severe enough to cause complete functional impairment. He was unable to protrude his tongue for more than ten seconds. We also noted partial bilateral ptosis, mild facial weakness, and complete ophthalmoplegia. The limbs were hypotonic, arreflexic with grade 3/5 power. He had severe proprioceptive loss at the wrists and ankles, vibration loss to the elbows and knees, and preserved pinprick and temperature sense. A week later, he developed acute respiratory distress requiring intubation.

On investigation, he had normal routine bloods, thyroid function, vitamin B12, and autoimmune screen, including antiGM1 and GQ1b IgG. IgM paraprotein was 6 g/L (5 g/L, August 2013). CSF was acellular, with normal glucose and protein. MRI brain was normal. Nerve conduction studies of the limbs showed absent bilateral median, ulnar, sural, and left radial sensory responses. F waves were delayed. Distal motor latencies were prolonged, with markedly reduced conduction velocities (Table 1). Unfortunately, we were unable to perform neurophysiological studies of the cranial nerves or facial EMG, because of the rapid trajectory of his deterioration. The patient was strongly positive for antiGD1a, GD1b, GT1b, GQ1b, GD3, and suphatides confirming CANOMAD (Table 2).

The patient was given 2 g/kg of IVIG over 2 days resulting in gradual improvement. By 6 weeks, he was mobilizing at premorbid level with no residual bulbar features. He has had monthly maintenance IVIG infusions (0.4 g/kg) for over twelve months without further exacerbations, even following major surgery.

## 3. Discussion

In the definitive 18-patient case series by Willison et al., patients with CANOMAD consistently suffered loss of vibration and proprioception sense, resulting in gait and upper limb ataxia and arreflexia with near normal limb power. On a background of slowly progressive symptoms, 13 of 18 patients had relapsing ocular, sensory or bulbar, occasionally motor, and rarely respiratory symptoms. Patients were typically male, with mean onset at 53 years and symptom duration of 13 years. Serologically, all 18 patients had high titre IgM antiganglioside antibodies. In fact, 16 patients had 3 or more antibodies targeting GD1b, GT1b, GQ1b, or GD3. Electrophysiological features varied. Usually patients had absent or markedly reduced sensory nerve action potential amplitudes, but some had motor conduction abnormalities. Nerve conduction studies were usually consistent with demyelination but some had axonal degeneration. CSF and neuroimaging studies were usually unremarkable [2]. Case reports show minor variations from this paradigm. For example, Sanvito and Rajabally describe two patients with optic nerve involvement [7].

As regards age, gender, chronic sensory ataxia, and relapsing brainstem symptoms, our patient is typical, as are his CSF studies, electrophysiology, and neuroimaging. His respiratory deterioration is rare, but not unique. However, his case is remarkable for involuntary facial movements, which are likely due to involvement of facial muscle 1a afferents or proprioceptive trigeminocerebellar fibres, similar to limb sensory ataxia [8]. In CANOMAD infections may trigger relapse [2]. In MFS, vaccinations, drugs, or malignancies are known triggers [3]. Food triggers, as in this case, are hitherto undescribed.

Importantly, this man was initially treated for MFS. MFS is a Guillain Barre variant, which presents with acute sensory ataxia and ophthalmoplegia related to antiGQ1b IgG. Symptoms usually resolve within ten weeks. Relapses are unusual [8]. The clinical presentation clearly overlaps markedly with CANOMAD and this case highlights the need for vigilance for recurrence and retesting of antiganglioside antibodies should recurrence occur.

The optimal treatment for CANOMAD is unclear. Case reports show variable responsiveness to IVIG, plasmapheresis, rituximab, and prednisone alone or in combination [2, 6, 9, 10]. In this patient, monthly IVIG therapy both induced remission and maintained the patient free from intermittent ocular, bulbar, and respiratory symptoms, albeit with baseline sensory ataxia. The use of IVIG to obtain sustained remission is supported by the case series by Attarian et al. [11].

In summary, we describe a unique case of CANOMAD, with involuntary facial movements and relapses triggered by food. This patient was initially misdiagnosed with MFS. We emphasise the importance of vigilance for recurrent neurological symptoms and the retesting of antiganglioside antibodies in these cases. Our patient responded remarkably to monthly IVIG, supporting the use of this therapy in both the acute and maintenance phases of treatment.

## Figures and Tables

**(a):** 

Motor nerve conduction			
Nerve	Latency	Amplitude	Conduction velocity
Median R (wrist)	8.9 ms	1.3 mV	
Median R (elbow)	18.5 ms	0.8 mV	24 m/s
Ulnar R (wrist)	4.4 ms	6.9 mV	
Ulnar R (below elbow)	10.4 ms	5.7 mV	38 m/s
Ulnar R (above elbow)	13.4 ms	3.3 mV	33 m/s
Tibial R (ankle)	6.2 ms	3.4 mV	
Tibial R (popliteal fossa)	23.1 ms	0.7 mV	24 m/s

**(b):** 

F waves		
Nerve	M latency	F latency
Ulnar right	4.4	42.7
Ulnar left	4	48
Tibial right	5.5	0
Tibial left	6.9	76.6

**(c):** 

Sensory nerve conduction	
Right median, right and left ulnar, left median, left radial, and right and left sural nerves (not recordable)	

**Table 2:** Serum antiglycolipid Antibody titres. Provided by Department of Neurological Sciences, Southern General Hospital, Glasgow.

Glycolipid	IgG	IgM	Normal ranges (IgG and IgM)
GM1	<500	<500	<500
GM2	<500	250	<500
GM3	<500	<500	<500
GA1	<500	140	<5000
GD1a	<500	>12500	<500
GD1b	<500	>12500	<500
GT1b	140	12400	<500
GQ1b	320	>12500	<500
GD3	<500	>12500	<500
Sulfatides	<10000	>12500	<10000
Globosides	<500	<500	<500
